# Clinical and Immunological Effects of p53-Targeting Vaccines

**DOI:** 10.3389/fcell.2021.762796

**Published:** 2021-11-03

**Authors:** Shan Zhou, Chunmei Fan, Zhaoyang Zeng, Ken H. Young, Yong Li

**Affiliations:** ^1^ Section of Epidemiology and Population Science, Department of Medicine, Baylor College of Medicine, Houston, TX, United States; ^2^ The Key Laboratory of Carcinogenesis and Cancer Invasion of the Chinese Ministry of Education, Cancer Research Institute and School of Basic Medicine, Central South University, Changsha, China; ^3^ Hematopathology Division, Department of Pathology, Duke University Medical Center, Durham, NC, United States

**Keywords:** p53, vaccine, Cancer, immunotherapy, T cell

## Abstract

Immunotherapy, including immune checkpoint blockade and chimeric antigen receptor T cells, is one of the most promising approaches to treat cancer. Vaccines have been effective in preventing cancers like liver cancer and cervical cancer with a viral etiology. Instead of preventing disease, therapeutic cancer vaccines mobilize the immune system to attack existing cancer. p53 is dysregulated in the majority of human cancers and is a highly promising target for cancer vaccines. Over twenty clinical trials have targeted p53 in malignant diseases using vaccines. In this work, we review the progress of vaccinations with p53 or its peptides as the antigens and summarize the clinical and immunological effects of p53-targeting vaccines from clinical trials. The delivery platforms include p53 peptides, viral vectors, and dendritic cells pulsed with short peptides or transduced by p53-encoding viruses. These studies shed light on the feasibility, safety, and clinical benefit of p53 vaccination in select groups of patients, implicating that p53-targeting vaccines warrant further investigations in experimental animals and human studies.

## Introduction

*TP53* gene encodes the transcription factor p53, one of the most important tumor suppressors. Under physiological conditions, p53 expression is tightly controlled and maintains a low level due to rapid degradation by the ubiquitin-mediated proteolysis. The E3 ubiquitin ligase MDM2 and its structural homolog MDMX (also known as MDM4) are the best known negative regulators of p53 (Manfredi, 2021). MDM2 polyubiquitylates p53 and results in proteasome-mediated degradation and monoubiquitylates p53 lead to export p53 out of the nucleus (Wu and Prives, 2018). Furthermore, MDM2 direct interacts with p53 to disrupt the transcriptional activity (Wu and Prives, 2018). Finally, MDM2 is a p53 target gene, thus creating an auto-regulatory feedback loop. MDMX has no ubiquitylation activity, but it binds p53 and inactivates it directly or heterodimerizes with MDM2 to aid MDM2 in p53 ubiquitylation (Wade et al., 2010; Karni-Schmidt et al., 2016; Yang et al., 2021). Under cellular stress, p53 becomes activated and stabilized to transcriptionally regulate target genes that are pivotal to various cellular processes, including cell cycle arrest, apoptosis, and DNA repair (Janic et al., 2018; Boutelle and Attardi, 2021).

## P53 in Cancer

Germline or somatic mutation in the *TP53* gene is frequently found in human cancers (Malkin et al., 1990; Hollstein et al., 1991). Missense mutations are the most common mutation type in cancer tissues, leading to mutant p53 accumulation in tumor cells (Petitjean et al., 2007; Mantovani et al., 2019). Mutations occur throughout the p53 protein but predominantly are located at exons 4–9 that encode the DNA binding domain, including six “hotspot” residues, namely R175, G245, R248, R249, R273, and R282 (Leroy et al., 2014; Bouaoun et al., 2016). Missense *TP53* mutations are classified as contact or structural mutations. Contact mutations, such as R248Q, R273H, and R273C, disrupt p53 DNA binding, resulting in loss of essential protein-DNA contacts. Structural mutations, such as R175H, G245S, Y220C, and R249S, destabilize the p53 structure and reduce its thermostability (Joerger and Fersht, 2007). Most p53 mutants not only lose wild-type (WT) p53 activity (i.e., loss of function [LOF]) but also obtain dominant-negative (DN) functions to antagonize the remaining WT p53 (Nakayama et al., 2020; Tang et al., 2020). Furthermore, many p53 mutants acquire gain of function (GOF) activity (Nakayama et al., 2020; Tang et al., 2020), though this is debated (Boettcher et al., 2019). Thus, mutant *TP53* acts as an oncogene that promotes tumor cells’ survival, proliferation, invasion, and metastasis. However, the *TP53* mutation rates differ significantly in anatomical tumor sites (Wang and Sun, 2017). A Pan-Cancer cohort showed *TP53* was the most frequently mutated gene (42% of samples); it is mutated in 95% serous ovarian cancer, but only in 2.2% renal clear cell carcinoma (Kandoth et al., 2013). The incidence of the LOF p53 mutations is associated with increased chemotherapy resistance and lower efficacy of anti-tumor agents (Keshelava et al., 2001). Overexpress WT p53 in tumor cells increase p53 protein level and lead to cell growth arrest or apoptosis (Ramqvist et al., 1993; McIlwrath et al., 1994). Furthermore, mouse models have demonstrated that the significance of p53 as a regulator of tumor suppression and therapy *in vivo* (Iwakuma and Lozano, 2007). p53 upregulates the expression of CDKN1A, BAX, PUMA, and NOXA, resulting in cell-cycle arrest, apoptosis, and senescence *in vivo* (Brady et al., 2011; Li et al., 2012).

p53 also plays a pivotal role in regulating inflammation in cancer through its activities in non-cancer cells. Lowe and colleagues showed that in the presence of chronic liver damage, ablation of a p53-dependent senescence program in hepatic stellate cells enhanced the transformation of adjacent epithelial cells into hepatocellular carcinoma by skewing macrophage polarization towards a tumor-promoting M2-state (Lujambio et al., 2013). Mice with a targeted deletion of p53 in myeloid cells selectively lost the Ly6c^+^CD103^+^ population and became unresponsive to immunotherapy and immunogenic chemotherapy, supporting that p53 drives differentiation of monocytic precursor cells into dendritic cells and macrophages for cross-presentation of tumor antigens (Sharma et al., 2018). MDM2 promoted T cell-mediated anti-tumor immunity by preventing c-Cbl-mediated STAT5 degradation; targeting the p53-MDM2 interaction with a pharmacological agent (APG-115) augmented MDM2 in T cells, boosted T cell immunity, and synergized with cancer immunotherapy (Zhou et al., 2021).

Multiple p53-targeting therapeutic strategies have been attempted. For tumors with WT p53, the approach is to suppress the interaction between p53 and MDM2/MDMX, inhibit the degradation of WT p53, and maintain the needed levels of p53 in cells, thus promote tumor suppression. For tumors with mutant p53, therapeutic agents are developed to reactivate mutant p53 or promote its degradation (Chen et al., 2021). Targeting the p53 signaling pathway has been extensively reviewed by a number of investigators (Hernández Borrero and El-Deiry, 2021; Huang, 2021; Liu et al., 2021; Salomao et al., 2021). At least theoretically, therapeutic restoration of inactivated tumor suppressors is more challenging than inhibiting an oncogenic target. Indeed, no therapy has successfully reactivated a mutated tumor suppressor in a clinical setting. Furthermore, p53 is an intracellular protein, making it inaccessible to antibodies.

## Cancer Immunotherapy

Cancer immunotherapy manipulates the immune system to recognize and destroy cancer cells. Therapeutic cancer vaccines are an exciting development in cancer immunotherapy by eliciting specific immune responses to tumor antigens. CD8^+^ cytotoxic T lymphocytes (CTLs) are preferred effector cells for anti-tumor immune responses (Hossain et al., 2021). CD8^+^ CTLs act as the key player in mediating tumor suppression through recognition of tumor-specific or associated antigens. T lymphocytes recognize antigens presented by antigen-presenting cells (APCs) in a major histocompatibility-restricted manner. Dendritic cells (DCs), discovered and characterized by Steinman and Cohn in 1973, are the most efficient APCs (Steinman and Cohn, 1973; Steinman and Cohn, 1974; Steinman et al., 1975). DCs participate in a variety of immunological processes, including initiating immune responses and sustaining effective T-cell-mediated anti-tumor immune responses (Marciscano and Anandasabapathy, 2021). DCs are classified into immature and mature according to their developmental stage. When immature DCs recognize, uptake, and cross-present the antigens released by tumor cells, they shift to secondary lymphoid organs, where they activate CD4^+^ T cells or CD8^+^ T cells to trigger specific CTLs responses against target cells (Wang et al., 2020a).

## Vaccines Targeting P53

Both WT and mutant p53 epitopes can be presented on the cell surface in the context of MHC I molecules by APCs for CD8^+^ T cell recognition (Houbiers et al., 1993). CTLs recognizing the WT p53_25–35_, p53_110–124_, p53_108–122_, p53_149–157_, and p53_264–272_ epitopes have been reported, and many are used for developing potentially broadly applicable cancer vaccines (Chikamatsu et al., 1999; Chikamatsu et al., 2003; Rojas et al., 2005; Ito et al., 2006). p53_110–124_ specific CD4^+^ T cells promote the generation and function of tumor-specific CD8^+^ CTLs (Chikamatsu et al., 2003). Short peptides from a mouse mutant p53 are recognized by CD4^+^ and CD8^+^ T cells, and vaccination with a mutant peptide emulsified in incomplete Freund’s adjuvant leads to tumor inhibition (Noguchi et al., 1994; Theobald et al., 1995). p53 mutations are associated with overexpression of mutant p53 in cancer cells, which may lead to the abnormal presentation of p53 peptides by APCs. p53_264–272_ or p53_149–157_ tetramer^+^ CD8^+^ CTLs have been detected in the circulation of head and neck squamous cell carcinomas (HNSCC) patients and negatively correlated with p53 expression in tumor tissues and tumor stage (Albers et al., 2018). These reports suggest that p53-specific CD8^+^ CTLs could eliminate tumor cells, as the immune system has the ability to recognize the p53 epitopes represented on the surface of cancer cells and APCs. Based on this evidence, a growing number of clinical trials targeting p53 have been conducted (Vermeij et al., 2011; DeLeo and Appella, 2020). This work comprehensively reviews these clinical trials with therapeutic vaccines against p53. Table 1 contains the clinical trial enrollment information such as vaccine platform, antigen type, and cancer type, and Table 2 provides the information on induced immune and clinical responses in cancer patients.

**TABLE 1:** Clinical trials with p53-targeting vaccines in human Cancers.

Author	Year	Phase	Vaccine platform	Antigen^a^ ^,^ ^b^	Disease	Patient no	Disease state	Previous treatment	Immunizations (x)
Kuball	2002	Pilot study	Recombinant adenovirus	WT FL p53	Urogenital, lung cancer, malignant schwannoma	6	Advanced disease	Unknown	4
Menon	2003	I/II	Recombinant canarypox virus	WT FL p53	Colorectal cancer	16	Metastatic disease	Chemotherapy/radiation therapy/other	3
Svane	2004	I	Short peptide-pulsed DC	WT p53 peptide	HLA-A2^+^ breast cancer	6	Metastatic disease	Chemotherapy/radiotherapy/endocrine	10
Lomas	2004	I	Short peptides plus GM-CSF	Short peptides derived from human anti-p53 (WT denatured) antibodies	Breast, colorectal, non-small-cell lung, renal, prostate, head- and neck, hemangiopericytoma, esophageal cancer	14	NED/metastatic /recurrent disease	Yes	4
Antonia	2006	I/II	Recombinant adenovirus-transduced DC	WT FL p53	Small cell lung cancer	29	Extensive/recurrent disease	Chemotherapy	3 or 6
Herrin	2007	II	Short peptide-pulsed DC	Short WT p53 peptide	HLA-A2^+^ ovarian cancer	21	Advanced/recurrent disease	Yes	>3
Svane	2007	II	Short peptide-pulsed DC	3 WT p53 peptides + 3 mutant p53 peptides (to enhance HLA-A2 binding	HLA-A2^+^ breast cancer	26	Metastatic	Regimens/endocrine	10
Leffers	2009	II	Long peptide	10 long peptides covering WT p53 (70–248)	Ovarian cancer	20	Recurrent disease	Surgery/chemotherapy	4
Speetjens	2009	I/II	Long peptide	10 long peptides covering WT p53 (70–248)	Colorectal cancer	10	Metastatic disease	Surgery/chemotherapy	2
Yoo	2009	II	Recombinant adenovirus	WT FL p53	Squamous Cell Carcinoma	13	Advanced disease	No	2
Trepiakas	2010	I/II	Short peptide-pulsed DC	Short peptides for p53 (mutated to enhance HLA-A2 binding), survivin, and telomerase	Melanoma	46	Metastatic disease	Chemotherapy	1–29
				Long peptide					
Rahma	2012	II	Short peptide pulsed-DC	Short WT p53 peptide	HLA-A2^+^ ovarian cancer	21	Advanced/recurrent disease	Yes	4
Vermeij	2012	II	Long peptide	10 long peptides covering WT p53 (70–248)	Ovarian cancer	12	Recurrent disease	Chemotherapy	4
Iclozan	2013	II	Recombinant adenovirus-transduced DC	WT FL p53	Small cell lung cancer	56	Advanced disease	Chemotherapy/radiotherapy	3
Zeestraten	2013	I/II	Long peptide plus IFN-α	10 long peptides covering WT p53 (70–248)	Colorectal cancer	11	Metastatic disease	Surgery/chemotherapy/radiotherapy	2
Hardwick	2014	I	Recombinant vaccinia Ankara virus	WT FL p53	Pancreatic cancer, colon cancer	12	Unresectable and chemotherapy-resistant disease	Chemotherapy/radiotherapy	3
Schuler	2014	I	Short peptide-pulsed DC	Short WT p53 peptides (mutated to enhance HLA-A2 binding)	HLA-A2^+^ HNSCC	16	Advanced disease	Surgery/chemotherapy	3
Dijkgraaf	2015	I/II	Long peptide	10 long peptides covering WT p53 (70–248)	Ovarian cancer	8	Platinum-resistant disease	Chemotherapy	2
Hardwick	2018	I	Recombinant vaccinia Ankara virus	WT FL p53	Ovarian cancer	12	Platinum-resistant disease	Chemotherapy	3
Soliman	2018	I/II	Recombinant adenovirus-transduced DC	WT FL p53	Breast, colon, gastric, lung, tongue, ovarian, chondrosarcoma cancer	194	Metastatic disease	Chemotherapy	4
Chiappori	2019	II	Recombinant adenovirus-transduced DC	WT FL p53	Small cell lung cancer	78	Recurrent disease	Chemotherapy	3
Chung	2019	I	Recombinant vaccinia Ankara virus	WT FL p53	Breast, pancreatic, hepatocellular, or head and neck cancer	11	Recurrent disease	Chemotherapy	3

a WT, wild-type.

b FL, full-length.

**TABLE 2:** Immune and clinical response p53-targeting vaccines.

Author	Year	Humoral response^a^	ELISpot	CD137 assay	p53-specific proliferation^b^	Treg frequency decrease	MDSC frequency decrease	Immunohistochemistry^c^	Clinical response^d^ ^,^ ^e^	Adverse events (Grade)
Kuball	2002	0/6	0/6	Not analyzed	Not analyzed	Not analyzed	Not analyzed	3/6 positive	4/6 SD, 2/6 PD	1
Menon	2003	Pre 7/15 post 10/15	Not analyzed	Not analyzed	Not analyzed	Not analyzed	Not analyzed	Not analyzed	1/16 SD, 15/16 PD	1/2
Svane	2004	Not analyzed	4/6 PR	Not analyzed	Not analyzed	Not analyzed	Not analyzed	3/6 positive	2/6 SD, 2/6 PD, 2/6 MR/UR	1/2
Lomas	2004	Pre 0/6 post 1/6	0/6 PR	Not analyzed	2/6 VIR	Not analyzed	Not analyzed	14/14 positive	Not analyzed	1/2
Antonia	2006	Pre 10/22 post 10/22	16/28 PR	Not analyzed	Not analyzed	No	Not analyzed	Not analyzed	1/29 PR, 7/29 SD, 21/29 PD	1/2
Herrin	2007	Not analyzed	14/20 PR	Not analyzed	Not analyzed	Not analyzed	Not analyzed	Not analyzed	Not analyzed	1–4
Svane	2007	Not analyzed	8/22 PR	Not analyzed	Not analyzed	Not analyzed	Not analyzed	11/26 positive	8/19 SD, 11/19 PD	1/2
Leffers	2009	Pre 8/20 post 9/20	18/18 PR	Not analyzed	14/17 PR	Not analyzed	Not analyzed	9/20 positive	2 SD, 18 PD	1/2
Speetjens	2009	Not analyzed	6/9 PR	2/10 CD4^+^ PR, 0/10 CD8^+^ PR	7/10 VIR	No	Not analyzed	7/10 positive	5/10 NED, 5/10 RD	1/2
Yoo	2009	Not analyzed	Not analyzed	Not analyzed	Not analyzed	Not analyzed	Not analyzed	Not analyzed	3/13 RD	1–4
Trepiakas	2010	Not analyzed	1/4 PR	Not analyzed	Not analyzed	No	Not analyzed	Not analyzed	11/36 SD	1/2
Rahma	2012	Not analyzed	14/20 PR	Not analyzed	Not analyzed	No	Not analyzed	Not analyzed	4/20 NED, 16/20 RD	1–4
Vermeij	2012	Not analyzed	7/8 PR	Not analyzed	5/8 VIR	No	Not analyzed	5/12 positive	2/10 SD, 8/10 PD	1/2
Iclozan	2013	Not analyzed	3/15 PR, 5/12 PR	Not analyzed	Not analyzed	No	No	Not analyzed	Not analyzed	Not reported
Zeestraten	2013	Pre 7/8 post 7/8	11/11 PR	Not analyzed	4/9 VIR	Not analyzed	Not analyzed	8/11 positive	Not analyzed	1/2
Hardwick	2014	Pre 0/5 post 5/5	6/6 PR	8/12 CD4^+^ PR, 10/12 CD8^+^ PR	Not analyzed	No	5/9	Not analyzed	Not analyzed	1/2
Schuler	2014	Not analyzed	4/16 PR, 11/16 PR (Tetramers)	Not analyzed	Not analyzed	12/15	Not analyzed	8/16 positive	13/16 NED	1/2
Dijkgraaf	2015	Not analyzed	8/8 PR	Not analyzed	0/8 VIR	No	No	Not analyzed	3/6 PD, 1/6 SD, 2/6 PR	1–4
Hardwick	2018	Not analyzed	Not analyzed	5/11 CD4^+^ PR, 6/11 CD8^+^ PR	Not analyzed	7/11	6/11	Not analyzed	3/11 SD, 1/6 PR	1–4
Soliman	2018	Not analyzed	7/23 PR	Not analyzed	Not analyzed	Not analyzed	Not analyzed	44/94 positive	4/39 SD	1–5
Chiappori	2019	Not analyzed	13/38 PR	Not analyzed	Not analyzed	Not analyzed	Not analyzed	Not analyzed	2/61 NED, 13/61 SD, 1/61 PR, 35/61 PD	1–3
Chung	2019	Not analyzed	Not analyzed	2/11 CD4^+^ PR, 2/11 CD8^+^ PR	Not analyzed	Not analyzed	Not analyzed	Not analyzed	3/11 SD, 6/11 RD	1–4

a Pre- and post-immunization levels of anti-p53-specific antibodies.

b p53-specific T-lymphocytes induced by immunizations. PR, positive response; VIR, vaccine-induced response.

c p53-staining of primary tumor samples.

d SD, stable disease; PD, progressive disease; MR, mixed response, UR, unconfirmed regression; PR, partial response; RD, recurrent disease; NED, no evidence of disease.

e All according to Response Evaluation Criteria in Solid Tumors.

### Peptide Vaccines

Lomas et al. conducted a phase I trial with up to four doses of a pool of eight short peptides derived from the complementarity determining regions of human anti-p53 antibodies (Lomas et al., 2004). In this trial, 14 patients with solid tumors were enrolled, and six received all four idiotypic vaccinations. The serum anti-vaccine antibodies were mainly IgG. One patient had increased titers of anti-p53 antibodies. Two patients showed responses in the thymidine proliferation assay to immunized peptides. In contrast to the proliferation assays, no patients had vaccine-specific, IFN-γ-secreting T cells as assessed by the enzyme-linked immune absorbent spot (ELISpot) assay (Lomas et al., 2004).

Leffers et al. conducted a phase II trial with a p53 synthetic long peptide (p53-SLP) vaccine in 20 ovarian cancer patients (Leffers et al., 2009). The p53-SLP vaccine contains 10 25–30 amino acid long peptides covering WT p53 from position 70–248. Before immunization, eight of 20 patients had p53 auto-antibodies associated with p53 expression in primary tumors. After immunization, nine presented p53-autoantibodies. Before immunization, responses against peptides included in the vaccine were present in three patients. After completing the immunization scheme, all patients had detected vaccine-induced IFN-γ producing p53-specific T-cells. These p53-specific IFN-γ T cells were CD4^+^CD8^−^. Two of the total 20 patients had stable disease as evaluated by CA-125 and computerized tomography (CT) and they had vaccine-induced p53-specific responses. Eighteen of 20 patients had clinical, biochemical, and/or radiographic evidence of progressive disease (Leffers et al., 2009). The authors concluded that the p53-SLP vaccine does not affect responses to secondary chemotherapy or survival and that p53-specific T cells do survive chemotherapy (Leffers et al., 2012).

The p53-SLP vaccine combined with cyclophosphamide therapy is evaluated by Vermeij et al. for treating patients with recurrent ovarian cancer in a single-arm phase II study (Vermeij et al., 2012). Twelve patients were administered four doses of the p53-SLP vaccine at a 3 week interval. Two days before each immunization, patients were treated with cyclophosphamide infusion. After four immunizations, seven of eight evaluable patients displayed vaccine-induced IFN-γ-producing p53-specific T cells, and five produced both T-helper 1 and T-helper-2 cytokines. The p53-SLP vaccine and cyclophosphamide combination therapy had no effect on T_reg_ cells. Two patients had stable disease as evaluated by serum CA-125 measurement and CT scan, and vaccine-induced p53-specific responses were present in both patients (Vermeij et al., 2012).

Speetjens et al. conducted a phase I/II trial with two doses of the p53-SLP vaccine in 10 metastatic colorectal cancer patients (Speetjens et al., 2009). Six of nine vaccinated patients with p53-SLP had p53-specific immune response detected by IFN-γ ELISpot. Furthermore, two showed detectable p53 specific CD3^+^CD4^+^CD137^+^ cell responses, none had CD3^+^CD8^+^CD137^+^ cell responses. One patient showed a p53-specific proliferative response before vaccination; seven of 10 patients displayed vaccine-induced p53-specific reactivity after vaccination. Vaccination has no effects on the induction of p53-specific T_reg_ cells. After vaccination, five had no evidence of disease, five showed recurrent disease (Speetjens et al., 2009).

In an ensuing phase I/II clinical trial, the combination of interferon IFN-α and p53-SLP was evaluated in 11 colorectal cancer patients (Zeestraten et al., 2013). The patients were treated with metastasectomy, chemotherapy, and/or radiofrequency ablation (RFA) for disease metastasis. p53-specific IgG antibody responses were detected in seven of eight patients who had serum samples from pre- and post-vaccination. In all patients, p53-SLP vaccination combined with IFN-α treatment-induced p53-specific T-cell responses. The toxicity of the combination was limited to Grade 1 or 2. After the two vaccinations, four of nine patients showed vaccine-induced proliferative responses (Zeestraten et al., 2013).

A phase I/II trial combining gemcitabine, IFN-α and the p53-SLP vaccine was conducted in patients with platinum-resistant ovarian cancer by Dijkgraaf et al. (2015). Patients were sequentially treated in three groups: the first three patients received gemcitabine alone, the following six patients received gemcitabine and IFN-α and the remaining six received gemcitabine, IFN-α, and additionally p53 SLP vaccine. Patients who received gemcitabine/IFN-α/p53 SLP treatment showed profound T-cell activation and increases in activated T-cell/T_reg_ cell ratios. All p53 SLP vaccinated patients showed detectable p53-specific T-cell responses (Dijkgraaf et al., 2015). Eleven Grade 3/4 adverse events were observed, most likely due to chemotherapy and/or IFN-α (Dijkgraaf et al., 2015).

From the above studies, we can conclude that the p53-SLP vaccine is safe and capable to induce p53-specific T-cell responses in patients treated for multiple cancers, yet improved survival is yet to come. Most studies are underpowered to demonstrate efficacy in the specific cancer population.

### Recombinant Viral Vaccines

Recombinant viral vaccines aim to use a live virus or attenuated virus to induce an immune response against the viral-encoded antigen (Nascimento and Leite, 2012). There are a number of viral platforms in vaccinations for many pathogens that have thwarted efforts towards control using conventional vaccine approaches (Ewer et al., 2016; Nasar et al., 2017), many of which are used to target p53 in cancer.

In a pilot clinical trial, six advanced-stage cancer patients were immunized with four doses of rAd/hup53 particles (Kuball et al., 2002). rAd/hup53 is a recombinant replication-defective adenoviral vector encoding human full-length WT p53, and it is capable of priming A2.1-restricted and hup53 epitope-specific CTLs *in vivo* but unable to induce p53-specific antibodies. After vaccinations, adenoviral backbone induced CD4^+^ T cells and CD8^+^ T cells in six and two patients, respectively. The treatment was well tolerated, yet no evidence for objective tumor responses was observed (Kuball et al., 2002).

The canarypox virus (ALVAC) is a well-characterized viral vector capable of infecting without replicating in mammalian cells. A phase I/II study was performed on 16 colorectal cancer patients with three intravenously injections of increasing dose of ALVAC encoding the human WT p53 gene (ALVAC-p53) at 3 week intervals (Menon et al., 2003). All patients had metastatic disease of p53-overexpressing colorectal cancer. Fever was the only vaccination-related adverse event. Before the vaccination, seven patients had IgG responses against p53; after vaccination, IgG responses against p53 were induced in three more patients (Menon et al., 2003). Following vaccinations, only one patient showed stable disease, while others showed progressive disease (Menon et al., 2003). No anaphylactic reaction or unwanted autoimmune reactions were observed (Menon et al., 2003).

Modified vaccinia virus Ankara (MVA) is a highly attenuated cytopathic strain replication-competent virus, a well-established vaccinia virus that the Food and Drug Administration has approved as a smallpox vaccine. In a phase I trial of p53MVA (an MVA virus carrying the WT p53 gene), 12 patients with refractory pancreatic and colon cancer were treated with three increasing doses of p53MVA every 3 weeks (Hardwick et al., 2014). Activation-induced CD137 expression is a common marker for antigen-triggered T cell responses. In this study, four patients had CD137^+^CD4^+^ T cells, and 10 had CD137^+^CD8^+^ T cells upon stimulation with the p53 peptide library. An MVA antibody neutralization assay showed that all patients had a low anti-MVA response before vaccination, whereas vaccination increased T cell reactivity and neutralizing activity against MVA. Patients with lower frequencies of PD1^+^ CD8^+^ T cells had greater p53-reactive CD8^+^ T cells after immunization, and antibody blockade of PD-1 *in vitro* increased the p53 immune responses (Hardwick et al., 2014). This first-in-human single-agent trial showed the p53MVA vaccine is well tolerated and immunogenic, but it showed no significant clinical responses.

Hardwick et al. conducted a dose de-escalating phase I trial of p53MVA vaccine in combination with the gemcitabine chemotherapy (Hardwick et al., 2018). Twelve patients with platinum-resistant ovarian cancer were enrolled in this trial and treated with gemcitabine before three p53MVA vaccinations. Five patients had CD137^+^ CD4^+^ T cells, and six had CD137^+^ CD8^+^ T cells after vaccination. In 11 patients evaluated for toxicity of the p53MVA/gemcitabine combination therapy, clinical outcome, and immunologic response, none had complete responses, three had stable disease, and one had a partial response on the second post-therapy CT scan (Hardwick et al., 2018).

Chung et al. conducted a phase I trial with the combination of p53MVA and pembrolizumab (anti-PD-1) to treat patients with advanced solid tumors (Chung et al., 2019). Eleven patients with advanced breast, pancreatic, hepatocellular, or head and neck cancer received up to three dose vaccines combined with pembrolizumab at a 3-week interval. They observed clinical responses in three patients who maintained stable disease for up to 49 weeks. Two of them showed increased p53 specific CD137^+^CD4^+^ and CD137^+^CD8^+^ T cells and upregulated multiple immune response genes (Chung et al., 2019).

Advexin (INGN 201, Ad5CMV-p53) is a replication-impaired adenoviral vector that carries the p53 gene under the cytomegalovirus (CMV) promoter and is a well-tolerated and efficacious treatment, both as a monotherapy and in combination with radiation and/or chemotherapy agents (Zhang et al., 1994). Yoo et al. conducted a phase II trial of surgery with perioperative INGN 201 gene therapy. Thirteen patients with advanced, resectable squamous cell carcinoma of the oral cavity and oropharynx were treated with INGN 201 along with surgery and chemoradiotherapy. After surgery, all patients received perioperative INGN 201 injections in the primary tumor bed and the ipsilateral neck. In addition, three patients received injections in the contralateral neck. All but three patients received chemoradiotherapy (Yoo et al., 2009). Of the 10 patients with evaluable data, two experienced Grade 4 adverse events and three died with observed relapses before death (Yoo et al., 2009). Overall, the estimate of 1 year progression-free survival was 92%, yet no definitive conclusion can be made with this small sample size.

### DCs Pulsed With p53 Peptides

Schuler et al. conducted a randomized phase I trial with p53 peptide-pulsed DCs in patients with HNSCC (Schuler et al., 2014). Both class I and class II peptides from p53 are used in this study: p53-sequences 149–157 with T150L and 264–272 with F270W are HLA-A2.1+ restricted (p53-I), and p53-sequence 110–124 are DR4+ restricted (p53-II). A T-helper [Th] tetanus toxoid peptide (Tt-II) is used as a control for p53-II. 16 HLA-A2.1^+^ patients were randomized into three arms: six in arm 1 (DCs with p53-I peptides), four in arm 2 (DCs with p53-I peptides + Tt-II peptide), and six in arm 3 (DCs with p53-I peptides + p53-II peptide). Vaccine-pulsed DCs were delivered to inguinal lymph nodes at the third time point. After vaccination, 12 of 15 patients showed decreased T_reg_ cells, and 13 had no evidence of disease in a median follow-up of 32 months. Eight patients had p53-positive tumors, but there was no difference in disease-free survival between patients with p53-positive versus p53-negative tumors (Schuler et al., 2014). There were no Grade 2–4 adverse events.

Svane et al. conducted a phase I trial of vaccination with p53 peptide-pulsed DCs in patients with advanced breast cancer (Svane et al., 2004). Six HLA-A2-associated p53 short peptides were used (3 WT and three were mutated to enhance HLA-A2 binding), along with and a pan-MHC class II peptide, PADRE. Nine patients received 10 immunizations with p53- and PADRE-peptide–pulsed autologous DCs. Before vaccination, two of them had T-cell reactivity against p53 peptides. After four or six vaccinations, four patients showed increased specific T-cell responses against p53 peptides. In three patients with vaccine-induced reactivity, T-cell responses were declined at late time intervals. Two patients maintained stable disease for more than 6 months (Svane et al., 2004).

A phase II trial of vaccination with the same p53 peptide-pulsed DCs for patients with advanced breast cancer was conducted by Svane et al. (2007). This phase II trial enrolled 26 patients with progressive metastatic breast cancer patients. Eight of 22 evaluated patients had p53-specific CTLs after immunization. p53 was frequently expressed in tumors from patients achieving stable disease. Five of six patients with stable disease expressed p53, whereas only six of 18 with progressive disease. Overall, among 19 patients available for first evaluation after six vaccinations, eight had stable disease, and 11 had progressive disease, supporting an effect of p53-specific vaccination (Svane et al., 2007).

Trepiakas et al. conducted a phase I/II trial with DCs pulsed with multiple tumor peptides from p53, survivin, and telomerase in 46 patients with malignant melanoma (Trepiakas et al., 2010). The p53 peptides (and the PADRE peptide) were the same as above. One out of four patients had increased lysate-specific IFN-γ response as detected by ELISpot, and six of 10 showed detectable antigen-specific T cell response as assessed by MHC multimer assays. After six vaccinations, compared to patients with progressive disease, patients with stable disease displayed significantly lower T_reg_ cells. Thirty-six patients had a clinical response: 11 had stable disease, six had continued stable disease after 16 weeks, and six had continued stable disease after 19 weeks (Trepiakas et al., 2010).

In an ensuing phase II study, metastatic melanoma patients were treated with p53 peptides-pulsed DC vaccination with interleukin-2, metronomic cyclophosphamide, and a Cox-2 inhibitor. The same six p53 peptides and the PADRE peptide were used. Among 28 patients evaluated: 16 had stable disease, and 12 had progressive disease (Ellebaek et al., 2012). The authors concluded that DC vaccination in combination with IL-2, cyclophosphamide, and the Cox-2 inhibitor was safe and tolerable, and a general increase in immune responses was observed upon fourth vaccination; however, a correlation between clinical benefit and a vaccine-induced T-cell response could not be determined (Ellebaek et al., 2012).

Herrin et al. conducted a randomized phase II trial with p53 vaccine to compare subcutaneous direct administration with intravenous peptide-pulsed DCs in high-risk ovarian cancer patients (Herrin et al., 2007). A single WT p53 epitope (264–272) with high HLA-A2.1 affinity was used for vaccination. Twenty-one patients were enrolled in this phase II study. On the subcutaneous arm, nine of 13 patients had an immunologic response. On the intravenous arm, five of seven had an immunologic response. Mean overall survival on the subcutaneous and intravenous arm is 70.4 and 72.9 months, respectively (Herrin et al., 2007).

### DCs Transduced With Virus

Antonia et al. tested a cancer vaccine based on adenovirus-transduced DCs (Ad.p53-DC) in a phase I/II study (Antonia et al., 2006). The virus expressed a full-length WT human p53. Twenty-nine patients with late-stage small cell lung cancer (SCLC) enrolled and received three vaccinations every 2 weeks. Ten patients had a detectable level of anti-p53 antibody before vaccination, and only three had a significantly increased anti-p53 antibodies level after immunization. p53-specific T cell responses were detected in 13 of 25 patients who underwent the IFN-γ ELISpot assay (Antonia et al., 2006). Among evaluated patients treated with this vaccine, one achieved a partial response, seven showed stable disease, and 21 developed progressive disease (Antonia et al., 2006).

Forkhead box protein P3 (FOXP3) expressing regulatory T (T_reg_) cells are a subset of CD4^+^ T cells with high immunosuppressive activity, which is critical cells for maintaining dominant self-tolerance and immune homeostasis (Togashi et al., 2019). T_reg_ cells exert their immunosuppressive activity through various cellular and humoral mechanisms, including cytotoxic T lymphocyte antigen 4 (CTLA-4)-mediated suppression of APCs, consumption of IL-2, and production of immune inhibitory cytokines and molecules (Spolski et al., 2018; Tekguc et al., 2021). T_reg_ cells can suppress anti-tumor immunity, and T_reg_ cells dysregulation is associated with a poor prognosis in human cancer patients (Wang and Ke, 2011; Saito et al., 2016). There are two types of antigens presented in tumor cells, including non-self-antigens, also known as neoantigens, derived from either oncogenic viral proteins or mutant proteins, and self-antigens, which are generated from highly or aberrantly expressed endogenous proteins. Self-antigens reactive CD8^+^ T cells exhibit an anergic phenotype owing to suppression by T_reg_ cells, while non-self-specific CD8^+^ T cells showed resistance to T_reg_ cells mediated suppression in humans (Maeda et al., 2014). Thus, T_reg_ cells exert more effective suppression in immune responses against self-antigens than non-self-antigens. Programmed cell death 1 (PD-1) is a negative regulator of T_reg_ cells as well as effector T cells, suggesting that PD-1 blockade enhances the suppressive function of T_reg_ cells (Kamada et al., 2019; Kumagai et al., 2020). The effects of p53-targeting vaccination on T_reg_ cells were estimated in this Ad.p53-DC phase I/II study (Antonia et al., 2006). Before or after vaccination, there was no significant change of these cells in healthy subjects and patients with SCLC, and no statistically significant link between the presence of these cells in the patients’ blood and p53-specific T cell responses (Antonia et al., 2006).

Myeloid-derived suppressor cells (MDSCs) are a heterogeneous population of cells. MDSCs are pathologically activated myeloid progenitors and immature myeloid cells with potent immunosuppressive activity (Hegde et al., 2021; Veglia et al., 2021). A number of studies have demonstrated that MDSCs are implicated in T cell suppression and are closely associated with poor clinical outcomes in cancer (Wang et al., 2020b; Imazeki et al., 2021). Mouse experiment also demonstrated that MDSC depletion with antibodies or different compounds could substantially improve anti-tumor immune responses to exert anti-tumor effects (Gabrilovich et al., 2012). Iclozan et al. conducted a randomized phase II trial in patients with late-stage SCLC using the Ad.p53-DC vaccine (Iclozan et al., 2013). Fifty-six patients were randomized into one of three arms: 18 to arm A (control), 19 to arm B (the Ad.p53-DC vaccine alone), and 19 to arm C (the Ad. p53-DC vaccine plus all-trans retinoic acid (ATRA). ATRA substantially decreases MDSC. Patients were administrated with the Ad.p53-DC vaccine three times at 2 week intervals. The Ad.p53-DC vaccine alone showed no effect on the frequency of MDSC and T_reg_, while the p53 vaccine combined with ATRA significantly decreased MDSCs (Iclozan et al., 2013). In addition, three patients in arm B had p53-specific immune response, and five in arm C had detectable p53 response (Iclozan et al., 2013). In a following article, Chiappori reported the clinical results of a randomized-controlled phase II trial of patients with recurrent SCLC with the same three arms (Chiappori et al., 2019). No immune response was detected in arm A (control), three of 15 patients showed positive immune responses in arm B, and 10 had positive immune response in arm C. In arm B, two patients maintained complete response, four had stable disease, 13 had progressive disease, and one had partial response. In arm C, nine patients had stable disease and 22 had progressive disease (Chiappori et al., 2019).

A phase-I/II study of the Ad. p53-DC vaccine in combination with indoximod in metastatic tumors were reported by Soliman et al. (2018). Forty-four patients with p53-positive by immunohistochemistry were enrolled in this trial. Seven of 23 patients had increased CD8^+^ T cell positive response, and six showed increased CD8^+^CD69^+^ T cells at week 3. During the vaccination period, no objective responses occurred; stable disease was observed in four patients at week 7 (Soliman et al., 2018). Overall, the Ad.p53-DC vaccine is safe and elicits immune responses, yet it fails to improve the overall response to chemotherapy.

## Conclusion

p53 is mutated in about half of all cancers and has attracted great interest in the development of cancer vaccines. An increasing number of studies on p53 vaccines, either peptide-, virus-, or DC-based for cancer immunotherapy, have been reported. Several vaccines have been tested in multiple clinical trials: p53-SLP, p53MVA, DCs with six p53 peptides and the PADRE peptide, and DCs transduced with Ad.p53. Key findings from these published clinical trials are summarized (Tables 1, 2). First, the p53 vaccines themselves are safe, *albeit* patients may have high-grade adverse events when they have adjunctive chemotherapy. Second, p53 vaccines elicit p53-specific immune responses. Third, current p53 vaccines do not improve patient survival to justify even a phase III trial, let alone approved to treat patients. Finally, current p53 vaccines are largely dependent on WT p53 full-length protein or peptides, which may circumvent the avidity of the CTLs due to self-tolerance (Theobald et al., 1997; Kuball et al., 2002). As vaccination technologies have unprecedented progress and successes during the COVID-19 pandemic, we call for further development of personalized p53-targeting vaccines with the following provocative questions. 1) Should we include antibodies in assays testing the B cell responses in addition to T cell activation? Mutant p53 is known to be released into the circulation of cancer patients (Sobhani et al., 2020). It is unclear whether the elicited antibodies from vaccines targeting mutant p53 in the serum or within the tumors offer therapeutic benefits. At a minimum, these antibodies may attract APCs to the tumor sites where mutant p53 antigens enrich. 2) Should we worry about autoimmune reactions targeting the endogenous WT p53 in physiologic tissues? Current p53 vaccines do not show widespread anaphylactic and autoimmune toxicities. It is notable that the R175H neoantigen is only presented 1.3–2.4 copies per cell in several tumor cell lines carrying the R175H mutation (Hsiue et al., 2021). It is unlikely that low endogenous WT p53 within normal tissues pose a serious challenge for T cell autoimmune response. 3) Will improved delivery methods help? mRNAs encapsulated in lipid nanoparticles (LNP) elicit about 5-fold neutralizing antibodies against the antigen (Spike) compared to adenoviral vectors (Khoury et al., 2021). mRNA-LNP may deliver mutant p53 better than outdated delivery approaches. 4) Will enhanced immunogenicity of p53 help? Structural changes in p53, via either stabilizing mutants (Boeckler et al., 2008) or adding self-assembling modules (He et al., 2021), could maximize the host’s p53 expression, presentation, and immunogenicity. 5) Shall we construct one vaccine for one p53 mutant? Each amino acid substitution in p53 may alter the p53 conformation differently (Wang and Fersht, 2015; Joerger and Fersht, 2016), so the final degradation products from one p53 mutant may differ considerably from another mutant or the WT p53 that is rarely overexpressed in cancer. The human T cell repertoire is not necessarily devoid of low- and even residual high-avidity p53-specific CTLs, yet self-tolerance certainly limits the number of high-avidity CTLs binding to MHC-restricted WT p53 peptides (Theobald et al., 1997; Kuball et al., 2002). Rational design of next-generation personalized p53 vaccines requires an in-depth understanding of mutant p53 structure and function, proteolysis, B and T cell elicitation, vaccine trafficking and retention, antigen expression and presentation, germinal center reactions, and self-tolerance. Such knowledge is essential to achieve the most effective precision vaccine candidates to be tested in clinical trials, in order to reduce cancer mortality from p53 mutation.
