# Corrigendum: Clinical and immunological effects of p53-targeting vaccines

**DOI:** 10.3389/fcell.2023.1267661

**Published:** 2023-08-02

**Authors:** Shan Zhou, Chunmei Fan, Zhaoyang Zeng, Ken H. Young, Yong Li

**Affiliations:** ^1^ Section of Epidemiology and Population Science, Department of Medicine, Baylor College of Medicine, Houston, TX, United States; ^2^ The Key Laboratory of Carcinogenesis and Cancer Invasion of the Chinese Ministry of Education, Cancer Research Institute and School of Basic Medicine, Central South University, Changsha, China; ^3^ Hematopathology Division, Department of Pathology, Duke University Medical Center, Durham, NC, United States

**Keywords:** p53, vaccine, Cancer, immunotherapy, T cell

In the published article, there was an error in **Affiliations** for Yong Li [1,2]. Instead of “[1,2],” it should be “[1]” (i.e., Yong Li is only affiliated with Baylor College of Medicine).

The authors apologize for this error and state that this does not change the scientific conclusions of the article in any way. The original article has been updated.

